# Design, Synthesis, and Morphological Behavior of Polymer Gel-Based Materials for Thermoelectric Devices: Recent Progress and Perspectives

**DOI:** 10.3390/gels11070508

**Published:** 2025-07-01

**Authors:** Md. Mahamudul Hasan Rumon, Mohammad Mizanur Rahman Khan, Md Khairul Amin

**Affiliations:** 1 Department of Chemistry, Indiana University of Bloomington, Bloomington, IN 47405, USA; mdhasa@iu.edu; 2 Department of Mechanical Engineering, Gachon University, 1342 Seongnam-daero, Sujeong-gu, Seongnam-si 13120, Gyeonggi-do, Republic of Korea; 3 School of Chemistry, University of Edinburgh, David Brewster Road, Edinburgh EH9 3FJ, UK; khairul.che@gmail.com

**Keywords:** synthesis, morphology, polymer gel, thermoelectric devices, conductivity

## Abstract

The current level of achievement in obtaining suitable polymer gel-based materials for efficient applications in thermoelectric devices is insufficient, although a substantial amount of research has already been performed. In this context, further investigations are necessary to design and synthesize polymer gel-based materials for ionic thermoelectric device applications. Polymer gel-based materials have attracted extensive consideration because of their multiple benefits, including easy processing, eco-friendly waste, and versatility, making them excellent materials for ionic thermoelectric devices. However, the design and synthesis of suitable polymer gel-based materials for ionic thermoelectric devices are still challenging areas of research. The surface morphological topography of prepared polymer gels is an important issue in thermoelectric device applications. In this review, significant approaches for the design and synthesis of polymer gel-based materials are discussed. This review may provide an important reference for upcoming perceptions on the design and synthesis of polymer gel materials for thermoelectric devices.

## 1. Introduction

The advancement of wearable electronics increasingly relies on materials that combine mechanical flexibility, electrical conductivity, biocompatibility, and non-toxicity [1,2,3]. Hydrogels in wearable electronics have gained considerable attention for their potential for health monitoring, human–machine interaction, and personalized medicine [4]. These systems require materials that are not only lightweight and mechanically flexible but also capable of responding to various physiological or environmental stimuli [5]. Traditionally, materials such as metals, ceramics, and rigid semiconductors have been used in sensor platforms, energy harvesters, and data transmission units [6]. However, these materials often suffer from limited stretchability, poor biocompatibility, and discomfort when interfaced with soft biological tissues. Even recent advances in flexible electronics based on organic semiconductors and composites are constrained by stability issues, limited conformability, or toxicity concerns [7,8]. In response to these challenges, hydrogels have emerged as a promising class of materials capable of addressing these limitations and offering new opportunities for skin-compatible and bioresponsive electronics [9].

Hydrogels are three-dimensional polymer networks that retain large amounts of water and exhibit soft, tissue-like mechanical properties [10,11]. Their high ionic conductivity, tunable chemistry, and ability to interact with biological environments make them uniquely suited for applications in bioelectronics. In particular, hydrogels offer excellent adhesion to human skin; however, they offer limited mechanical similarities with tissues and responsiveness to temperature, pressure, and biochemical cues [12]. These features are crucial for the development of next-generation thermoelectric devices that aim to harvest low-grade thermal energy from the human body or ambient surroundings. However, conventional hydrogels used in thermoelectric systems often exhibit low ion mobility, weak mechanical robustness, and unstable performance under thermal or mechanical stress [13,14]. Overcoming these limitations requires new material design strategies that enhance ionic conductivity, improve structural integrity, and enable consistent operation in dynamic environments.

Recent advances in hydrogel engineering have begun to address these challenges by introducing materials with hierarchical architectures, phase-separated networks, and specialized doping to modulate ionic pathways [15,16,17]. These developments have led to significant improvements in thermoelectric performance, enabling devices that are stretchable, skin-conforming, and capable of continuous energy harvesting from body heat. Notable contributions from research groups, such as Zhenan Bao’s team at Stanford, have demonstrated hydrogel-based thermoelectric materials with Seebeck coefficients with enhanced charge and power densities suitable for self-powered wearables [18]. Building on this progress, our review aims to provide a comprehensive analysis of the roles hydrogels play in thermoelectric systems. We focus on material design, structure–property relationships, device performance metrics, and emerging strategies to push the boundaries of hydrogel-based energy harvesting toward practical, scalable applications.

## 2. Principles of Hydrogel-Based Thermoelectric

Hydrogel-based thermoelectric materials can be broadly grouped into three functional categories, each defined by a distinct mechanism for thermal-to-electrical energy conversion [1,19,20,21,22]. These include systems that rely on ion migration, those that operate through redox processes, and composite platforms that integrate inorganic thermoelectric elements within a soft hydrogel framework [23]. In ion migration systems, hydrogels function as ionic conductors when exposed to a temperature gradient [24,25]. The generated thermoelectric voltage results from the unequal movement of cations and anions, which is governed by differences in their charge, size, mobility, and interactions with the hydrated polymeric environment. The efficiency of this ionic thermodiffusion process depends strongly on factors such as ion concentration, polymer chain dynamics, porosity, and the degree of hydration. Systems enriched with highly mobile ions tend to exhibit stronger ionic conductivity and larger thermoelectric potentials.

The second class of hydrogel-based devices utilizes redox-active species dissolved within the hydrogel matrix [26]. In these systems, the thermoelectric response is driven by spatially separated redox reactions across a temperature gradient [27]. Typically, oxidation occurs at the hotter electrode, while reduction takes place at the cooler side, creating a directional flow of electrons through an external circuit. Electron transfer is coupled with the migration of counterions within the hydrogel to preserve charge balance. The overall voltage output is determined by the entropy difference between the oxidized and reduced states. Reversible redox couples such as ferricyanide and ferrocene derivatives are frequently employed because of their well-defined electrochemical behavior and thermal responsiveness [28].

**Figure 1:**
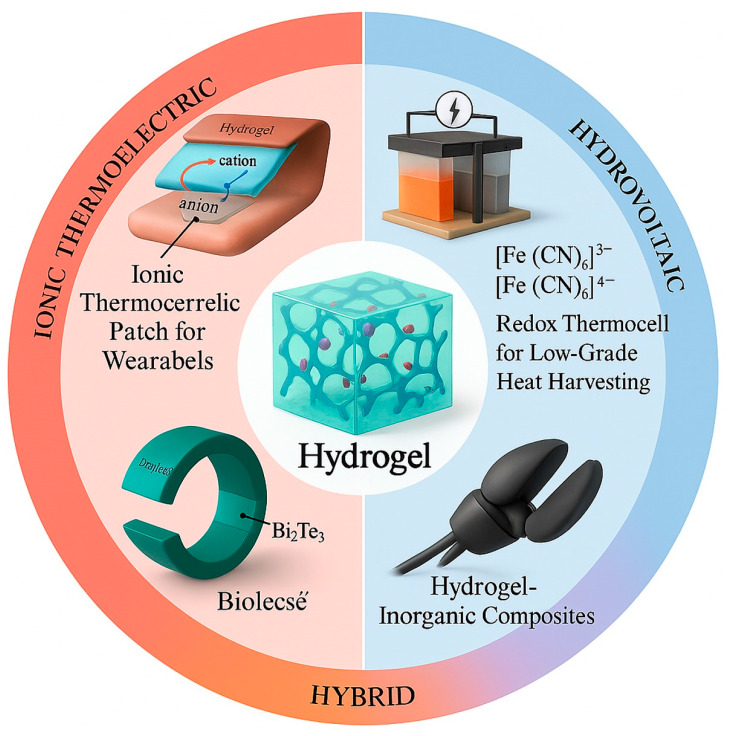
Overview of hydrogel-based energy systems: ionic thermoelectric patches, redox thermocells with [Fe(CN)_6_]^3−^/[Fe(CN)_6_]^4−^, and hybrid composites for enhanced energy harvesting.

The third category involves the incorporation of inorganic thermoelectric materials such as bismuth telluride or lead telluride into a hydrogel scaffold [29]. While the inorganic fillers offer high thermoelectric conversion efficiency through electronic charge carriers, the hydrogel matrix provides flexibility, enhanced processability, and improved mechanical compatibility with wearable systems [20,30,31]. These composite materials are carefully engineered to balance electrical performance with physical adaptability, making them attractive candidates for soft and conformal energy harvesting devices. Together, these three categories represent the foundational design strategies for hydrogel-based thermoelectric materials (Figure 1) [32,33]. Understanding the distinct roles of ionic mobility, redox thermodynamics, and composite integration provides a versatile platform for tailoring the next-generation energy conversion materials suited for bio-integrated and flexible electronics [31,34].

Several pioneering studies have demonstrated the potential of ionic thermoelectric (i-TE) patches through innovative material designs. Zhou et al. first introduced quasi-solid-state i-TE gels based on the thermoelectric effect, specifically using a PVA–Fe(CN)_6_^4−/3−^(H_2_O) system, achieving a thermopower of 1.21 mV K^−1^ [35]. In the same year, a significant thermoelectric response was observed in polystyrene sulfonic acid (PSSH) under highly humid conditions, where proton (H⁺) diffusion along the temperature gradient resulted in a thermopower of 8 mV K^−1^ [36]. Subsequently, in 2019, Crispin and co-workers reported a tunable i-TE gel system comprising PVDF-HFP and EMIM:TFSI, where the incorporation of neutral polyethylene glycol (PEG) shifted the dominant thermodiffusion ions from anions to cations. This ion-selective transport led to a reversal of thermopower from −4 mV K^−1^ to +14 mV K^−1^ [37]. Building on this progress, the authors’ group reported a benchmark i-TE gel in 2020 composed of gelatin, KCl, and Fe(CN)_6_^4−/3−^, which leveraged the synergistic contribution of thermodiffusion and thermoelectric effects to attain a remarkable thermopower of 17 mV K^−1^ [38].

**Figure 2:**
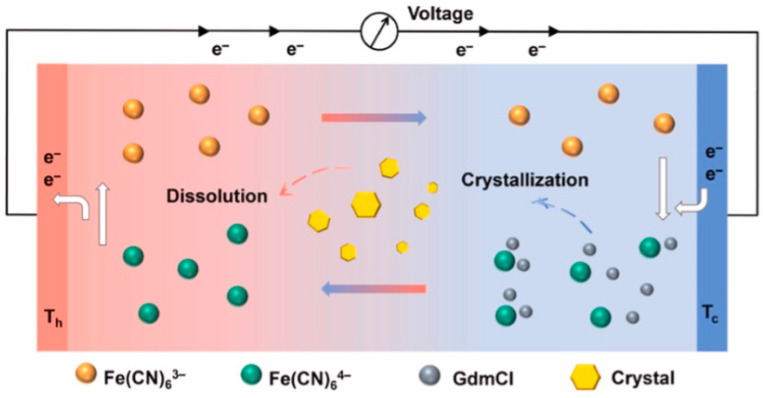
Schematic representations of different ionic thermoelectric mechanisms. The figure is adopted from Ref. [43].

Thermogalvanic hydrogels constitute an emerging class of soft materials capable of converting thermal gradients into electrical energy through the thermoelectric effect [31]. This process involves the generation of an electromotive force when a temperature difference is applied across a redox-active electrolyte. A typical hydrogel-based thermocell comprises two electrodes immersed in a redox-containing hydrogel electrolyte connected via an external electrical circuit (Figure 2). Upon imposing a thermal gradient between the electrodes, redox reactions are thermodynamically driven; oxidation occurs preferentially at the hotter electrode, while reduction proceeds at the cooler one [39]. The resulting ionic species redistribute through mechanisms such as diffusion, convection, and ionic migration, thereby closing the electrochemical loop and sustaining a continuous current. As long as the redox species remain chemically stable and unconsumed, this electrochemical cycle can, in principle, persist indefinitely. The combination of intrinsic flexibility, ionic conductivity, and sustainable redox cycling positions thermoelectric hydrogels as promising candidates for next-generation energy harvesting and storage systems, offering a viable pathway toward scalable and renewable power solutions [40,41,42].

## 3. Different Hydrogels for Thermoelectrics and Their Key Properties

Thermoelectric generators (TEGs) have emerged as promising power sources for wearable electronics because of their ability to harvest energy from body heat and environmental temperature gradients [44,45,46]. In this context, hydrogels play a pivotal role in the architecture of TEGs, not only providing flexibility and biocompatibility but also contributing directly to thermal and charge transport [22,47,48]. Their incorporation into TEG systems has significantly expanded, with diverse functionalities demonstrated depending on their chemical composition, structure, and intended thermoelectric mechanism [32].

This section explores the material design strategies and functional roles of various hydrogels in TEDs, focusing on how specific hydrogel chemistries influence performance, stretchability, and integration. Their versatility has enabled multiple roles in TEDs, ranging from active thermoelectric legs to flexible electrodes and thermal management layers.

Over the past few years, hydrogel-based materials have gained substantial attention because of their relatively high Seebeck coefficients, often in the range of tens of millivolts per kelvin [14]. This thermoelectric potential, combined with their compatibility with diverse synthesis techniques, has positioned them as strong candidates for use in flexible and wearable TEDs [19,31,49]. In particular, their soft and porous nature makes them ideal for harvesting body heat, promoting ion mobility, enabling signal coupling, and providing mechanical stability under deformation [3,50]. To further optimize the output power, mechanical resilience, and thermal response, it is essential to understand the heat harvesting behavior of p- and n-type hydrogel legs, their coupling with heat sinks, and the interfacial performance of integrated electrodes [51].

Hydrogels used in thermoelectrics typically function as solid–liquid hybrid electrolytes, facilitating the movement of both ions and, in some cases, electronic charges [52,53]. Compared with traditional liquid electrolytes, hydrogels offer several advantages: reduced leakage risk, greater mechanical integrity, and improved compatibility with wearable devices. Their soft, hydrated structure mimics biological tissue, making them ideal for on-body applications [54,55]. Most hydrogel electrolytes possess both flexibility and stretchability. These properties are essential for wearable TEDs that undergo repetitive bending, twisting, or stretching during use. The ion-conducting hydrogels are often engineered to include continuous channels or networks that guide ion migration, enabling consistent performance even under deformation. When a thermal gradient is applied across the hydrogel, mobile species such as sodium, lithium, chloride, or protons migrate from the hot region to the cold region [56]. This ion redistribution results in a measurable voltage. The degree of voltage generation is determined by the difference in mobility between the ionic species. Therefore, improving the thermoelectric power factor (S^2^σ), which depends on both the Seebeck coefficient (S) and electrical conductivity (σ), requires maximizing this mobility disparity [57]. A summary of polymer gel-based materials and their thermoelectric performances are listed in Table 1.

It is important to note that, while the S^2^σ metric is widely used to evaluate traditional thermoelectric materials, its application to ionogels is limited [52,78]. Ionogels often exhibit time-dependent fluctuations in conductivity due to water loss, ionic redistribution, or material aging [79,80]. These dynamic characteristics make steady-state performance metrics less accurate. Consequently, alternative evaluation frameworks may be needed to more precisely assess the reliability and efficiency of hydrogel-based TEDs over time.

**Figure 3:**
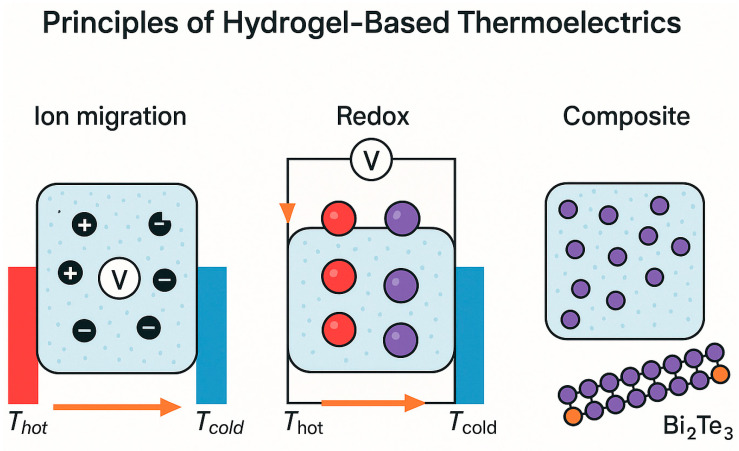
Schematic illustration of the working principles of hydrogel-based thermoelectrics. Three main mechanisms are depicted: (a) ion migration, where thermally driven ionic diffusion generates a voltage; (b) redox-based thermocells, where a temperature gradient drives reversible redox reactions; (c) composite systems, where conductive fillers such as Bi_2_Te_3_ enable electronic transport within the hydrogel matrix.

In systems relying on redox-active electrolytes, the hydrogel matrix plays a dual role by facilitating redox reactions and enabling charge transport [81,82]. For instance, when a temperature difference is applied across the electrodes, redox species such as ferricyanide undergo oxidation at the hot electrode and reduction at the cold electrode, leading to net electron flow through the external circuit (Figure 3) [83,84,85]. Simultaneously, ion movement within the hydrogel ensures charge neutrality. The generated Seebeck voltage in such cases arises from entropy differences of redox couples, and maximizing this entropy difference is key to improving overall energy conversion efficiency [86].

**Figure 4:**
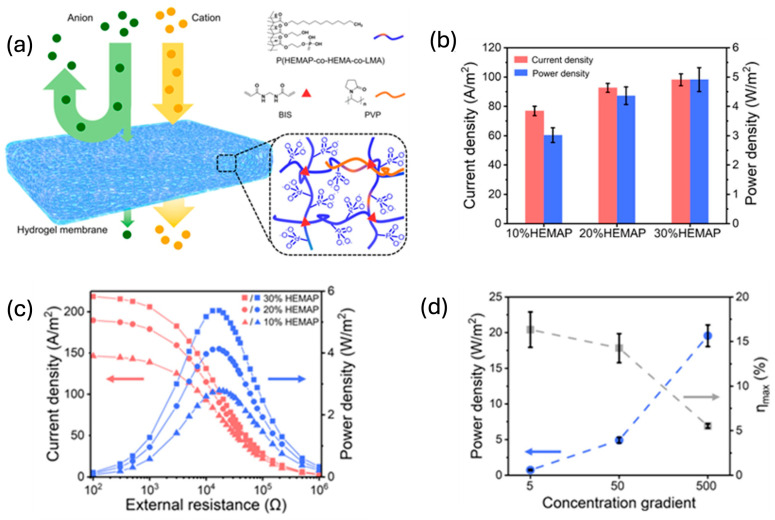
Schematic illustration and performance characteristics of three-dimensional interconnected HEMAP hydrogel membranes for osmotic energy harvesting. (**a**) Schematic representation of a space-charged HEMAP hydrogel membrane featuring an interconnected nanoporous architecture that facilitates enhanced ion transport and exhibits selective ion permeability. The membrane is integrated into a hydrogel-based power generator designed for harvesting osmotic energy. (**b**) Measured current densities and power densities of hydrogel membranes with varying HEMAP content. (**c**) Statistical analysis of current and power densities, with error bars indicating standard deviation. (**d**) Power densities and corresponding energy conversion efficiencies of the optimized hydrogel membrane under different salinity gradients. The figure is adopted from Ref. [91].

Hydrogels also exhibit relatively high thermal conductivity compared with air, primarily because of their high water content [47]. This property allows them to serve as effective thermal absorbers or buffers in TEDs, facilitating uniform heat distribution and preventing localized overheating. Their high specific heat capacity enables thermal regulation, and in some cases, water evaporation from the hydrogel matrix provides additional cooling [87,88]. For hydrogel-based heat sinks, the ability to absorb and release moisture from the environment enhances this passive cooling effect [89,90]. Furthermore, the three-dimensional porous structure of hydrogels supports efficient ion transport between electrodes [91]. The presence of interconnected pores and a hydrated environment maintains high ion mobility. This architecture also preserves hydration of the electrolyte, stabilizing performance over extended operation [92,93]. Combined with their inherent softness and adaptability, hydrogels are ideally suited for use in deformable and wearable electronics. A recent study conducted by Chen et al. [91] introduced a phosphate-functionalized hydrogel membrane, synthesized via photopolymerization of 2-hydroxyethyl methacrylate phosphate (HEMAP), which features a three-dimensional network of interconnected nanopores and intrinsic space charges. This architecture enables efficient, selective ion transport and supports a high-power density of 5.38 watts per square meter when applied to osmotic energy conversion. Mechanistic insights from both experimental and computational analyses emphasize the critical role of the porous network in regulating ion flux and enhancing charge-selective transport. Beyond energy harvesting, the structural versatility of this membrane also lends itself to applications in desalination and biosensing (Figure 4).

Despite such progress, current hydrogel-based thermoelectric materials face persistent challenges. Conductive polymer hydrogels, while mechanically flexible and processable, often exhibit suboptimal thermoelectric performance and limited long-term stability. Overcoming these limitations requires new strategies that simultaneously enhance ionic mobility, mechanical robustness, and environmental resilience [94]. In contrast, inorganic nanomaterial-reinforced hydrogels offer high Seebeck coefficients but may compromise mechanical softness or biocompatibility [94,95,96]. Additionally, the potential toxicity of some nanofillers poses challenges for wearable or implantable applications.

Hybrid hydrogel systems that combine organic and inorganic phases often struggle with interface compatibility and fabrication complexity [97]. Carbon-based hydrogels, including those incorporating graphene or carbon nanotubes, provide high conductivity but often underperform in thermopower and face scalability issues [98]. Ionic liquid-based hydrogels can offer tunable conductivity and structure but sometimes suffer from high viscosity, processing challenges, and cost [99,100]. Finally, purely polymeric hydrogels, while easy to fabricate and highly flexible, generally exhibit lower thermoelectric performance.

These trade-offs highlight the importance of tailoring hydrogel materials to specific device requirements [101,102]. Continued advances in composite design, crosslinking chemistry, and multifunctional integration will be essential for addressing these limitations and enabling broader adoption of hydrogel-based thermoelectric technologies.

### 3.1. Strategies for Improving Thermoelectric Properties in Hydrogel-Based Thermocells: Enhancing Thermopower

Despite significant promises, the broad adoption of hydrogel-based thermocells remains constrained by their relatively low power output, limited energy conversion efficiency, and modest long-term operational stability [103,104]. These limitations have driven active research focused on enhancing thermoelectric performance through molecular-level tuning, electrolyte engineering, and interfacial optimization [105]. The thermoelectric performance of thermocells is governed by thermopower, which reflects the entropy difference between redox species during electrochemical transformation [106]. This entropy change mainly stems from variations in solvation structures and the concentration distribution of ions near the electrodes. Enhancing either contribution, by tuning solvation shell organization or establishing a pronounced ion concentration difference, can significantly improve the voltage generated [107]. As a result, careful selection of redox couples and the design of the surrounding electrolyte environment are crucial for achieving high-efficiency ionic thermoelectric systems.

Improving the thermoelectric properties of hydrogel systems involves enhancing their intrinsic ionic conductivity and thermopower through targeted chemical and structural modifications [108]. Approaches such as optimizing the type and concentration of ionic species, introducing highly conductive ionic liquids, and engineering hydrogel polymer networks to facilitate efficient ion transport have shown significant promise [109]. Concurrently, adjusting the hydrogel’s crosslinking density and pore architecture enables better ion mobility, directly influencing thermoelectric efficiency [24]. Additionally, incorporating functionalized additives or nanoparticles can further boost the Seebeck coefficient by introducing controlled ionic interactions or selective ionic transport pathways [110]. Collectively, these strategies not only strengthen the fundamental thermoelectric properties of hydrogels but also broaden their practical applicability in sustainable energy harvesting and wearable bioelectronics.

In parallel with these material-level improvements, effective signal acquisition remains a major challenge in large-area sensing platforms [111]. Systems designed to collect data from extensive arrays of flexible sensors must meet several demanding criteria [112]. These include the elimination of unwanted signal overlap between adjacent sensing units, strong signal clarity, compact and efficient wiring, minimal energy use, rapid response, limited data size for transmission, low thermal generation, and reasonable processing demands for real-time analysis [113].

Two commonly used architectures are employed for reading signals from such sensor arrays [114]. The passive matrix system uses a grid formed by intersecting rows and columns, with sensing elements located at the crossings. While this design is relatively simple and compatible with scalable fabrication, it is susceptible to interference from stray currents that can distort the output [115]. Addressing this issue through complex circuitry may reduce reliability, especially as the total number of sensing points increases. The active matrix approach offers a more refined solution by placing switching components, such as transistors or diodes, directly at each sensing site [116]. This configuration allows each sensor to be addressed individually, which greatly reduces interference and simplifies the wiring layout. In addition, transistors can function as amplifiers at the sensing location, enhancing signal strength before transmission [117]. Originally developed for visual display technology, these systems are now widely used in flexible electronics and sensing devices that demand precision and large-scale integration [118].

Transistors serve essential functions in sensor arrays by enabling signal readout, conditioning, and local processing [119]. Their electrical characteristics directly influence the overall signal fidelity, energy efficiency, and reliability of the sensing matrix [120]. Recent efforts in this area have focused on developing stretchable transistor platforms that can match or approach the performance of their rigid counterparts [121,122,123]. To realize this, key targets include enhancing switching speed, reducing operating voltage, and achieving stable electrical output under mechanical strain [124]. In addition, minimizing power consumption remains a central requirement for large-area and wearable applications [125,126]. Beyond conventional designs, alternative operational mechanisms are being explored to further elevate transistor performance. One notable example is the use of subthreshold Schottky barrier thin film transistors, which can operate at very low power levels while delivering high signal amplification [127]. These transistors represent a promising direction for next-generation sensing electronics that demand both mechanical adaptability and energy-efficient computation.

Solvation entropy can be modulated by incorporating functional additives that reorganize or disrupt the solvent environment surrounding redox-active species [107]. These additives perturb the hydrogen bonding network or alter the dielectric properties of the local solvation shell, thereby increasing molecular disorder and the associated entropy. Redox couples possessing high absolute charges, such as the [Fe(CN)_6_]^3−^ and [Fe(CN)_6_]^4−^ pair, display a pronounced thermopower, significantly exceeding that of the I^−^ and I_3_^−^ couple. This enhancement arises from stronger electrostatic interactions and greater reorganization energy during redox transitions.

**Figure 5:**
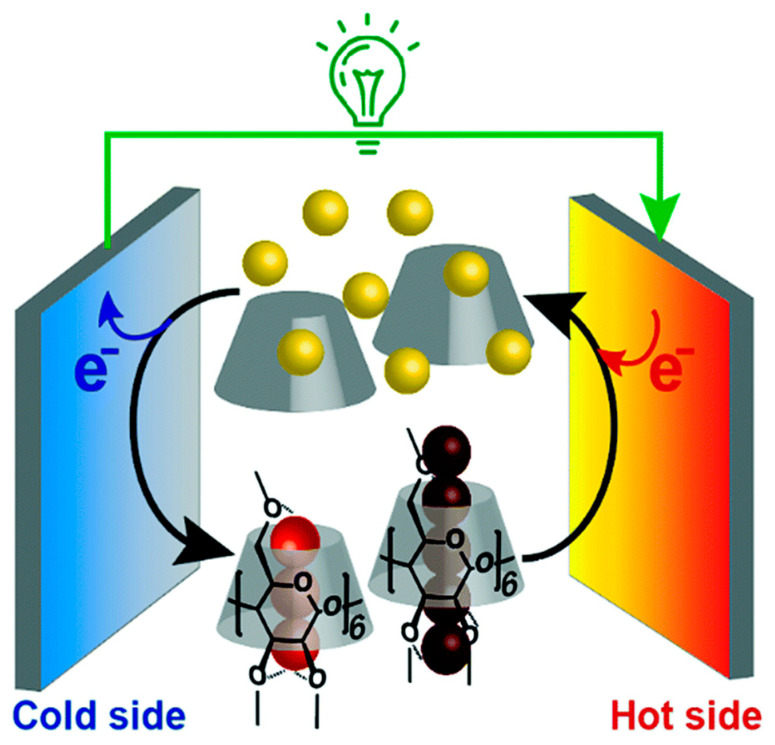
Schematic illustration of a supramolecular thermocell comprising I^−^ (yellow spheres), I_3_^−^ (trio of red spheres), I_5_^−^ (five connected dark red spheres), and Me18-α-cyclodextrin (Me18-α-CD; gray cone-shaped cylinder). The figure is adopted from Ref. [128].

Liang et al. demonstrated that hexakis(2,3,6-tri-O-methyl)-α-cyclodextrin (Me18-α-CD) serves as an effective host molecule for I^−^/I_3_^−^-based thermocells [128]. The aqueous Me18-α-CD/iodide solution exhibited excellent stability without precipitation, even in the presence of supporting electrolytes. This system achieved a Seebeck coefficient of 1.92 mV K^−1^, the highest reported for homogeneous I^−^/I_3_^−^ thermocells to date (Figure 5). Notably, an unusual non-stoichiometric interaction between Me18-α-CD and I_3_^−^ was observed, leading to the formation of a Me18-α-CD–pentaiodide (I_5_^−^) complex. This marks the first confirmation of I₅^−^ formation in aqueous solution, as previous reports have only identified this species in crystalline form. These findings highlight the importance of rational host design in enhancing thermoelectric performance in redox-based systems.

Solvent composition also plays a pivotal role. The inclusion of organic solvents with varying donor numbers influences the electronic density and size of the solvation shell [129]. Since entropy is inversely proportional to structural order, solvents that expand or destabilize the solvation environment elevate the thermoelectric response [130]. Notably, the use of mixed aqueous–organic media has enabled the development of high-entropy electrolytes, with thermopower values reaching up to 2.65 millivolts per kelvin. For example, the integration of organic co-solvents into hydrogels based on the ferricyanide and ferrocyanide redox couple resulted in a thermopower increase from 1.27 to 2.30 mV/K [131]. These systems were successfully incorporated into self-powered flexible sensors capable of detecting human motion with high fidelity and responsiveness.

Correspondingly, researchers have explored the enhancement of thermopower through deliberate control of the concentration gradient [132]. In typical thermocells, concentration gradients decay over time because of diffusion-driven equilibration, nullifying the driving force for thermoelectric generation. However, innovative approaches have demonstrated that spatial confinement and molecular recognition can stabilize non-equilibrium states. One such example involves the use of methylcellulose to trap triiodide ions near the hot electrode and release them at the cold electrode, thereby maintaining an asymmetric ion distribution that augments and even reverses the thermopower.

Han et al. report thermally induced polarization switching and ultrahigh n-type and p-type thermopowers by incorporating methylcellulose (MC) and KCl into the I^−^/I_3_^−^ redox electrolyte [133]. MC, a biocompatible, low-cost, and temperature-responsive polymer, undergoes a reversible transition between hydrophilic and hydrophobic states. Above its gelation temperature, MC exhibits hydrophobic interactions with I_3_^−^ ions, reducing their concentration near the hot electrode and reversing redox polarity. As a result, polarization switching from n-type to p-type occurs when the hot electrode surpasses the gelation threshold [133].

The binary electrolyte system (I^−^/I_3_^−^ + 2 wt% MC) yields enhanced thermopowers of −1.32 mV K^−1^ (n-type) and 1.48 mV K^−1^ (p-type), significantly exceeding the −0.71 mV K^−1^ of the pristine I^−^/I_3_^−^ electrolyte. Remarkably, the addition of KCl further amplifies this effect: the optimized ternary electrolyte (I^−^/I_3_^−^ + 2 wt% MC + 0.3 M KCl) delivers record-high thermopowers of −8.18 mV K^−1^ (n-type) and 9.62 mV K^−1^ (p-type), an order of magnitude greater than the unmodified system and among the highest reported for thermoelectric electrolytes. Temperature-dependent measurements reveal a transition temperature (Tₜᵣₐ) near 56 °C, corresponding closely with the MC gelation temperature (57 °C) as determined by differential scanning calorimetry, confirming the role of MC gelation in polarization switching.

Zhou et al. [134] implemented a host–guest system using alpha cyclodextrin and potassium chloride to modulate the local concentration of triiodide. The host selectively sequestered triiodide at the cold electrode, shifting the equilibrium toward further oxidation. Upon heating, the complex dissociated, increasing the hot-side triiodide content and promoting reduction. This dynamic interplay resulted in a large and stable concentration asymmetry, boosting the thermopower from 0.86 to 1.97 mV/K [134].

An additional strategy for magnifying the entropy and concentration difference involves the selective crystallization of redox ions. Guanidinium ions, known for their chaotropic character, were used by Yu and collaborators to induce cold-side crystallization of the [Fe(CN)_6_]^4−^ anion [135]. These thermosensitive crystals dissolved at the hot electrode, generating a sustained concentration differential. Experimental and computational studies confirmed that, at 293 K, nearly all [Fe(CN)_6_]^4−^ crystallized, reducing its availability for redox cycling. In contrast, at 343 K, the crystals fully dissolved, reestablishing redox activity. The resulting concentration gradient elevated the thermopower from 1.4 to 3.73 mV/K. Furthermore, the presence of solid-phase crystals suppressed thermal convection, reducing parasitic heat flow and increasing overall device efficiency. The system achieved a relative Carnot efficiency of 11.1%. In conventional liquid thermocells (LTCs), the concentration gradient (ΔCr) is thermodynamically unstable and naturally relaxes to a homogeneous equilibrium state, where ΔCr approaches zero in a 0.4 M K_3_Fe(CN)_6_/K_4_Fe(CN)_6_ aqueous electrolyte. Consequently, the Seebeck effect in such systems is solely entropy-driven (ΔS). Specifically, Fe(CN)_6_^4−^, possessing lower solvation entropy, is oxidized at the hot electrode to Fe(CN)_6_^3−^, which exhibits higher solvation entropy. The resulting electron flow through the external circuit is consumed at the cold electrode via the reverse reduction reaction. In contrast, this article demonstrates an enhanced Seebeck effect driven by both ΔS and ΔCr through the introduction of guanidinium (Gdm⁺) cations, which selectively induce Fe(CN)_6_^4−^ crystallization. Due to its higher charge density, Fe(CN)_6_^4−^ interacts more strongly with Gdm⁺ than Fe(CN)_6_^3−^, leading to cold-side crystallization and subsequent hot-side dissolution. This thermally reversible crystallization results in a low Fe(CN)_6_^4−^/Fe(CN)_6_^3−^ ratio near the cold electrode (~0.02 at 293 K) and a high ratio near the hot electrode (~0.94 at 343 K), establishing a pronounced ΔCr. As a result, both redox reactions are locally enhanced, intensifying the voltage output. The dual-gradient design is a thermosensitive crystallization–boosted LTC (TC-LTC), which achieves significant thermopower amplification via the synergistic effects of ΔS and ΔCr.

Building on this principle, Liu et al. [64] combined temperature-induced crystallization with mechanical deformation to develop a stretchable polyvinyl alcohol-based thermocell. This quasi-solid material exhibited exceptional thermoelectric properties, with a thermopower of 6.5 mV/K and a power density of 1969 mW/m^2^K^2^. These values surpass most previously reported flexible thermoelectric devices, underscoring the potential of crystallization-driven thermoelectrics.

### 3.2. Improving Ionic Conductivity

The ionic conductivity of hydrogel thermocells remains several orders of magnitude lower than that of inorganic thermoelectric materials [136,137]. This low conductivity imposes significant resistance to ion transport and constrains the maximum achievable power output. While increasing the concentration of redox-active ions can improve conductivity, it often raises the viscosity of the electrolyte, impairing mass transport and reducing overall efficiency [138,139,140]. Moreover, for some redox systems, elevated concentrations can suppress thermopower. For instance, the thermopower of the I^−^ and I_3_^−^ system decreases dramatically as ion concentration increases from 0.01 to 2 molar, with losses exceeding 400 percent [138,141]. To address these limitations, recent advances have focused on modifying the structure and composition of electrodes to facilitate ion and electron transport [142,143,144,145]. One successful strategy involves the fabrication of three-dimensional porous electrodes that enhance surface area and electrolyte contact [146]. Im et al. developed platinum nanoparticle-coated carbon nanotube aerogels that significantly increased charge transfer efficiency, achieving a conversion efficiency of 3.95 percent [147].

**Figure 6:**
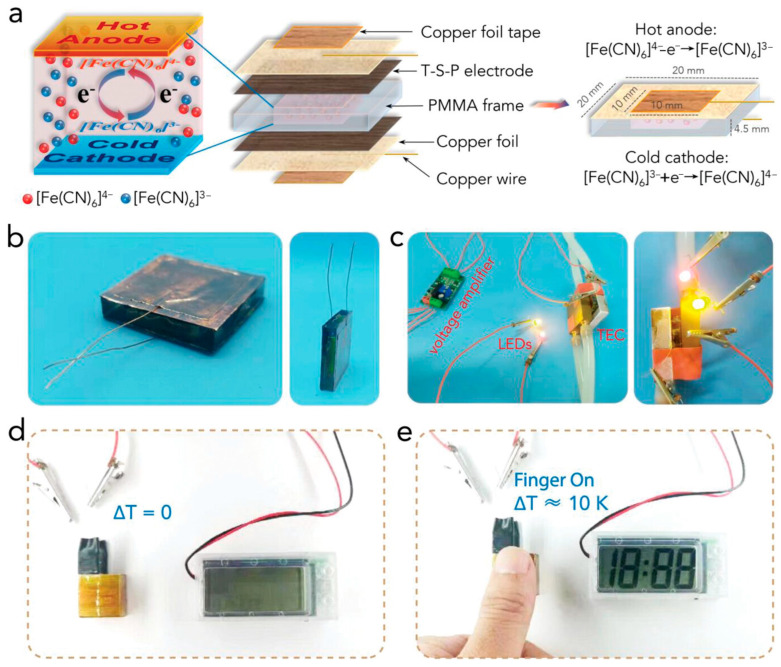
(**a**) Schematics of the configuration and size information of the square TEC. (**b**) Optical images of the as-fabricated square TEC. (**c**) Demonstration of using the square TEC to light up two LED bulbs under the temperature difference of 30 K with the assistance of a voltage amplifier. (**d**,**e**) Demonstration of using the square TEC to power an electronic timer by harvesting human body heat. The timer was immediately powered on once the thumb touched one side of the square TEC. The applied temperature difference between the human body and the ambient environment was ≈10 K. The figure is adopted from [148].

A more sophisticated electrode architecture was reported by Wei and coworkers [148], who engineered a multilayer system comprising MXene nanosheets, carbon nanotubes, and polyaniline. This hierarchical composite exhibited high electrochemical activity because of its synergistic structure. The MXene layers provided rapid electron conduction, while the polyaniline and nanotube network supported efficient ion diffusion. Under a temperature difference of 40 kelvin, the system delivered an outstanding power density of 13.15 milliwatts per square centimeter. To demonstrate practical applicability, a compact square thermoelectric cell (TEC) was fabricated using flexible T-S-P film electrodes. As illustrated in Figure 6a, the device features a polymethyl methacrylate (PMMA) frame as the electrolyte reservoir and copper foils as current collectors. The active region, comprising the electrode and electrolyte layers, measures 10 × 10 × 4.5 mm^3^, while the overall device dimensions are 20 × 20 × 4.5 mm^3^. Copper wires were connected to the collectors for external circuitry. Top- and side-view images of the assembled TEC are shown in Figure 6b. In contrast to bulkier 3D aerogel-based TECs, the film-based design significantly reduces device volume while maintaining efficient mass transport and redox activity. When subjected to a temperature gradient of 30 K and integrated with a voltage amplifier, the TEC successfully powered two LED bulbs (Figure 6c). Furthermore, the device effectively harvested low-grade body heat to activate an electronic timer within 3 s of contact with a fingertip, driven by a modest ~10 K temperature difference (Figure 6d,e), underscoring its promise for wearable energy harvesting applications. Collectively, these strategies highlight the central importance of interface design in thermoelectric devices. Enhancing electrode–electrolyte coupling and expanding the accessible reaction surface can dramatically reduce interfacial resistance, enabling higher current densities and improved energy harvesting performance. These developments position hydrogel-based thermocells as a promising platform for flexible, sustainable, and wearable thermoelectric technologies [148].

## 4. Gel Matrix and Its Influence on Ionic Thermoelectric Conversion

**Figure 7:**
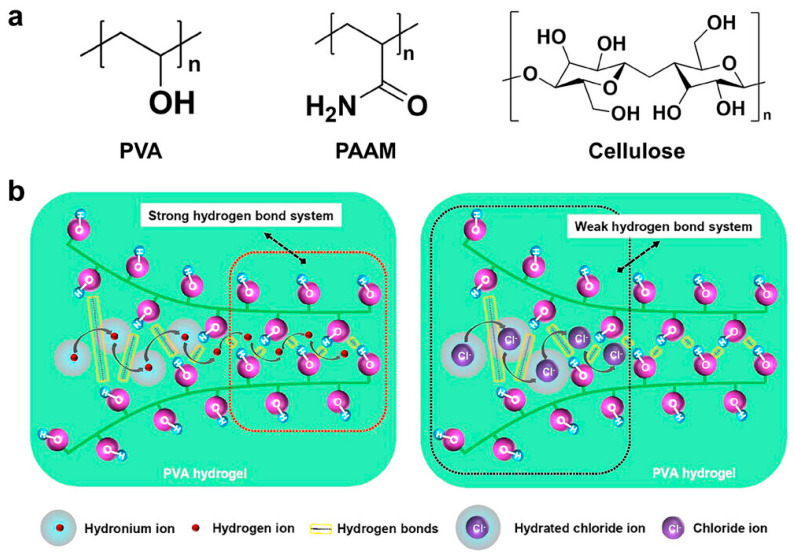
Representative polymer matrix systems. (**a**) Molecular structures of PVA, PAM, and cellulose. (**b**) Conceptual framework illustrating a thermoelectric device enhanced by proton conduction within a hydrogen bond-rich network. Reproduced with permission from Ref. [61]. Copyright 2022, American Chemical Society.

The gel matrix is a critical component in thermally chargeable capacitors (TCCs), primarily because the interactions between mobile ions and the polymer network significantly influence the overall performance of ionic thermoelectric (i-TE) systems [149]. Ideally, the selection of gel matrices should consider key attributes, such as mechanical durability, structural flexibility, and water retention capacity. Given that this review emphasizes recent developments in i-TE materials, relevant mechanical parameters reported in the current literature are summarized in this section. For a broader discussion covering mechanical features including elasticity, healing ability, and durability, readers are referred to comprehensive reviews by Xu et al. [150], Zhang et al. [33], and Li et al. [151]. To date, various types of polymer networks have been explored as gel matrices, including synthetic polymers such as PVA, PAAM, and biopolymers such as cellulose [61,152,153,154]. Chen et al. reported a PVA hydrogel system in which ionic thermopower arises from the selective transport of H⁺ across regions with varying degrees of crystallinity [61]. Upon infiltration of an inorganic acid into the physically crosslinked PVA network, the resulting hydrogel functions as an ionic conductor, exhibiting an exceptional thermopower of 38.20 mVK^−1^. This value exceeds more than twice the previously recorded maximum for hydrogen ion-based thermoelectric materials. The relevant molecular structures are presented in Figure 7 [61]. Additional matrices such as gelatin, PVDF-HFP, and waterborne polyurethane (WPU) are also discussed in the subsequent sections.

### 4.1. Polyvinyl Alcohol

PVA is a synthetic polymer featuring a polyethylene backbone and pendant hydroxyl groups, offering strong hydrophilicity [155]. This structure facilitates water absorption and provides a conducive environment for ion transport through its three-dimensional network. Two common fabrication methods are employed to produce PVA hydrogels: (i) chemical crosslinking, which introduces covalent bonds between polymer chains, and (ii) freeze–thaw cycling, which forms physical networks via hydrogen bonding and chain entanglement [49,156].

PVA hydrogels fabricated by freeze–thaw methods typically incorporate nanocrystalline regions that affect ionic mobility and thermoelectric behavior [157,158]. For example, Chen et al. developed a freeze–thawed PVA-HCl hydrogel in which the crystallinity was modified by a room-temperature drying and stretching protocol [61]. The resulting material exhibited anisotropic Seebeck coefficients, with a value of 38.20 mV K^−1^ along the tensile direction and 13.11 mVK^−1^ perpendicular to it. Their findings also showed that the hydrogen bond length within the crystalline PVA ranges from approximately 266 to 275 pm, while the chloride ion has a diameter of 362 pm.

Although PVA hydrogels typically function as p-type TCCs due to preferential cation migration, they can be transformed into n-type systems through specific post-processing treatments that alter ion–polymer interactions. Chen et al. reported that when using NaOH as the ionic source, a PVA hydrogel formed via freeze–thawing could be converted into an n-type TCC [59]. A heat treatment at 100 °C, followed by swelling, enabled the formation of strong coordination structures between PVA and Na⁺ ions. This structural arrangement disrupted the hydration shells around Na⁺, enhancing coordination and promoting n-type behavior. The resulting material exhibited a negative Seebeck coefficient reaching −37.61 mV K^−1^. These insights highlight the structural tunability of PVA for different thermoelectric polarities.

**Figure 8:**
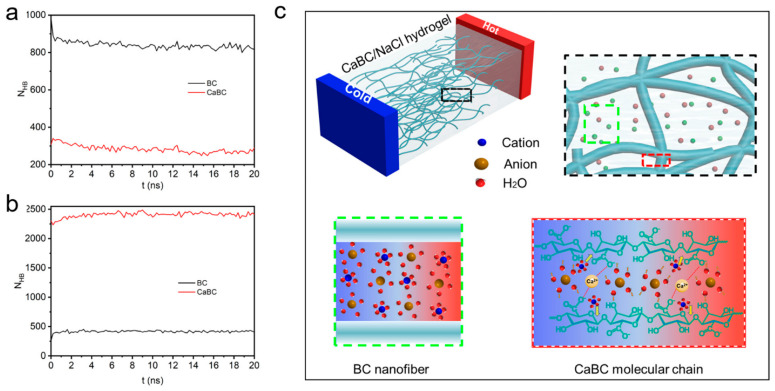
Ion transport and thermal diffusion in CaBC hydrogels. (**a**,**b**) The number of hydrogen bonds over simulation time: (**a**) between cellulose chains and (**b**) between cellulose and water, indicating weakened chain interactions and enhanced hydration. (**c**) A schematic of ion thermal diffusion between nanofibers and molecular chains in the CaBC/NaCl hydrogel. The figure is adopted with permission from Ref. [154]. Copyright 2022, American Chemical Society.

Wu et al. proposed a physical model to elucidate how ion–polymer interactions influence the thermoelectric behavior of ionic thermoelectric (i-TE) materials (Figure 8) [154]. It is well established that ions can modulate local hydrogen bonding through kosmotropic (structure-promoting) or chaotropic (structure-disrupting) effects. As illustrated in Figure 8a, the hydrogen bonding between CaBC polymer chains is markedly reduced compared with pristine BC chains, indicating a weakening of intermolecular interactions. This reduction likely results in expanded chain spacing, consistent with molecular simulation outcomes, thereby facilitating ion insertion into the CaBC matrix. Furthermore, Figure 8b reveals a pronounced increase in hydrogen bonding between water molecules and the CaBC chains, which may, in turn, disrupt the hydration shells of the absorbed ions. In such hydrogels, the mechanisms governing thermal diffusion differ between cations and anions. Cation transport is primarily governed by their affinity to polar groups on the polymer backbone, largely dictated by electronegativity. In contrast, anion mobility is predominantly influenced by the chaotropic effect, which alters the Gibbs free energy landscape (Figure 8c).

### 4.2. Polyacrylamide

PAM is composed of a polyethylene backbone substituted with amide groups (–CONH_2_) on alternating carbon atoms [159]. The hydrogel networks are generally synthesized through photo-initiated or thermally initiated polymerization of acrylamide (AM) monomers, often using N,N′-methylene bisacrylamide (MBAA) as a crosslinker [160]. PAM hydrogels are widely recognized for their excellent water retention, biocompatibility, and biodegradability. When used in composite structures, such as double-network hydrogels, they demonstrate significant improvements in mechanical strength and toughness. In a study by Yang et al., a composite hydrogel combining sodium carboxymethyl cellulose (CMC-Na) and PAAM was employed as an electrolyte, with NaCl serving as the ionic conductor [161]. This system exhibited a maximum Seebeck coefficient of 17.1 mV K^−1^ and maintained mechanical integrity under a stress of 0.71 MPa with a strain tolerance of 235%. Similarly, Huo et al. reported a three-dimensional composite structure based on chitosan, polyacrylamide, and silicon dioxide (PACS), doped with 1 M Li-TFSI electrolyte [152]. This hydrogel achieved a Seebeck coefficient of 25 mV K^−1^ and withstood 95 kPa of stress at a strain of 1298%. These results establish PAAM hydrogels as mechanically robust platforms for high-performance thermoelectric materials.

### 4.3. Cellulose

Cellulose is a linear natural polymer composed of D-glucose units linked by β-1,4-glycosidic bonds [50,162]. As the primary structural material in plant cell walls, cellulose offers excellent mechanical strength, thermal stability, and chemical resistance. Its surface is rich in hydroxyl groups and negatively charged sites, making it particularly suitable for coordinating with multivalent metal cations.

Wu et al. designed a calcium ion-coordinated bacterial cellulose hydrogel (CaBC), in which the calcium ions expanded the spacing between cellulose chains, thereby promoting ion migration along the cellulose framework. This system, when immersed in a NaCl solution, yielded a high Cl^−^ diffusion coefficient and achieved a Seebeck coefficient of −27.2 mV K^−1^ and an ionic conductivity of 204.2 mS cm^−1^. In another work, Chen et al. developed a natural cellulose hydrogel coordinated with Zn^2^⁺ ions and utilized 1-allyl-3-methylimidazolium chloride ([AMIM]Cl) as the ionic medium [96]. This material delivered a Seebeck coefficient of approximately −3.06 mV K^−1^. It also demonstrated outstanding mechanical properties, showing a 1500% increase in elastic modulus, a 1000% enhancement in toughness, and a tensile strength of 4.46 MPa. Chen et al. developed a high-performance cellulose-based ionogel (CZ ionogel) by doping ZnCl_2_ into a cellulose–ionic liquid–water system, achieving exceptional strength (4.46 MPa), high conductivity (67.43 mS cm^−1^), excellent transparency (94%), and freezing resistance (−103 °C) [163]. Zn^2^⁺ ions coordinate with cellulose via carboxyl and hydroxyl groups, forming Zn^2^⁺–cellulose interactions, dynamic hydrogen bonds, and ion–dipole interactions. These synergistic effects yield a robust network with ~15- and ~10-fold improvements in modulus and toughness over the undoped ionogel.

The confined structure selectively promotes Cl^−^ transport while restricting [AMIM]⁺ mobility, resulting in a significant negative thermovoltage (−3.06 mV K^−1^) and n-type thermoelectric behavior. Simulations confirm Cl^−^ as the dominant charge carrier. This sustainable, mechanically strong, and highly conductive ionogel overcomes conventional trade-offs, advancing the design of next-generation n-type ionic thermoelectric materials [163].

### 4.4. Other Polymer Matrices

Several other polymers have been utilized as gel matrices in ionic thermoelectric systems. Gelatin, a natural biopolymer, is valued for its biocompatibility, water solubility, and low processing cost. Han et al. first demonstrated an i-TE device using gelatin as the polymer host in 2020 [164]. By exploiting both thermoelectric and thermodiffusive effects, a combined Seebeck coefficient of 17.0 mV K^−1^ was achieved using the [Fe(CN)_6_]^3−^/^4−^ redox couple with KCl salts.

**Figure 9:**
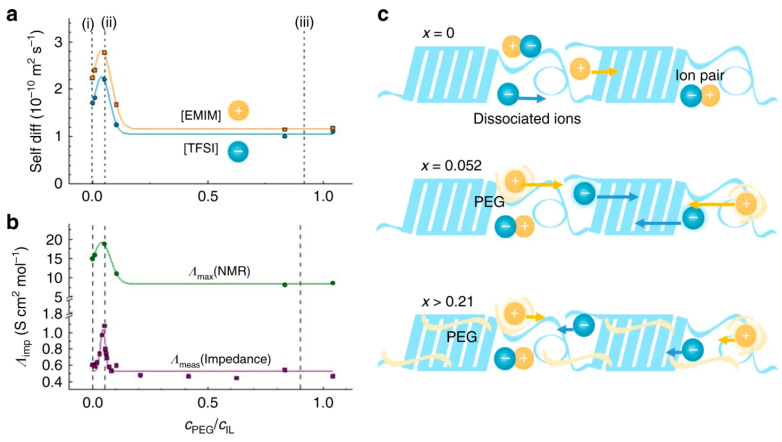
Self-diffusion behavior and ionic conductivity of polymer gels with varying PEG content. (**a**) Self-diffusion coefficients of [EMIM]⁺ and [TFSI]^−^ as a function of the molar concentration ratio of PEG to ionic liquid (c_PEG/c_IL), measured by pulsed field gradient NMR. (**b**) Molar conductivity derived from impedance spectroscopy (Λ_imp) and estimated using the Nernst–Einstein relation from NMR data (Λ_NMR), plotted against c_PEG/c_IL. (**c**) Schematic illustration of interactions among ions, PEG chains, and the polymer matrix within the gel. The figure is adopted from Ref. [37].

Zhou et al. further examined the effect of doping levels on the thermoelectric performance of a gelatin-based ionogel system containing 1-ethyl-3-methylimidazolium acetate ([EMIM][OAC]) [165]. The introduction of acetate salts such as lithium, sodium, potassium, cesium, ammonium, and nickel improved the ionic Seebeck coefficients at low doping levels. Among these, sodium acetate produced optimal performance, yielding a Seebeck coefficient of 37.3 mV K^−1^, an ionic conductivity of 12.3 mS cm^−1^, and an ionic ZT value of 2.1. Synthetic polymers, such as PVDF-based systems, have also shown promise because of their high crystallinity and chemical resistance. PVDF-HFP copolymers, when combined with ionic liquids, have demonstrated effective thermoelectric behavior. Zhao et al. first reported a PVDF-HFP-based TCC in 2019 [37]. By incorporating polyethylene glycol (PEG) into a PVDF-HFP and [EMIM][TFSI] ionogel, the thermodiffusing ion species shifted, allowing modulation of the Seebeck polarity. The effect of non-ionic PEG content on ion dynamics and ionic conductivity was evaluated by analyzing the self-diffusion coefficients of cations (D⁺) and anions (D^−^) using PFG-NMR spectroscopy (Figure 9). Both D⁺ and D^−^ increased with low PEG content (cPEG/cIL up to 0.052), suggesting enhanced ion mobility. However, at higher PEG concentrations (0.052 < cPEG/cIL < 0.21), the diffusion coefficients declined and eventually plateaued beyond cPEG/cIL = 0.21. This non-monotonic behavior likely reflects changes in the local microviscosity of the polymer network, although direct measurement remains challenging in solid-like gel systems [37]. Li et al. later developed a fiber-shaped TCC using PVDF-HFP and [EMIM][DCA] [76]. The inclusion of ethanol and Na-TFSI improved ion mobility and enhanced the difference in thermodiffusion between cations and anions, resulting in a Seebeck coefficient of 22.9 mV K^−1^ and an ionic conductivity of 17.5 mS cm^−1^.

WPU-based polymers have also attracted attention. Fang et al. reported a transparent and stretchable WPU and [EMIM][DCA] system in 2020, achieving a Seebeck coefficient of 34.5 mV K^−1^ and ionic conductivity of 8.4 mS cm^−1^ [166]. The specific interactions between WPU and anions restricted anion mobility, producing a net positive Seebeck coefficient. The same group introduced WPU-based eutectogels using choline chloride and ethylene glycol as a deep eutectic solvent [167]. These materials, free from ionic liquids, exhibited a Seebeck coefficient of 19.5 mV K^−1^ and maintained a conductivity of 8.4 mS cm^−1^, offering a sustainable and low-toxicity alternative.

## 5. Mechanistic Insights into Hydrogel Degradation

To ensure long-term performance and reliability of hydrogels in wearable and biomedical applications, a comprehensive understanding of their degradation mechanisms is essential [168]. Hydrogel degradation typically proceeds through three primary mechanistic pathways: physical, chemical, and biological, each dictated by the material’s composition, crosslinking density, and operational environment.

Physical degradation arises from mechanical and environmental stressors. Repetitive deformation (e.g., stretching and compression), swelling–deswelling cycles, and exposure to temperature or humidity gradients induce fatigue and microstructural failure [169]. These effects are particularly pronounced in mechanically dynamic environments, such as skin-interfaced wearables.

Chemical degradation encompasses hydrolysis, oxidation, and photolysis [170]. Hydrolytically labile linkages such as esters, anhydrides, and amides undergo nucleophilic cleavage in aqueous media, reducing molecular weight and disrupting network integrity [171]. Oxidative degradation, initiated by reactive oxygen species (ROS) or atmospheric oxygen, targets electron-rich functionalities (e.g., thioethers and phenols), leading to irreversible chain scission. In light-exposed systems, photodegradation of chromophoric units may also occur, especially under UV or visible irradiation, compromising structural stability.

Biological degradation involves enzymatic cleavage by naturally occurring hydrolases, including esterases, proteases, and lysozymes. This pathway is particularly relevant for biodegradable hydrogels and is strongly influenced by enzyme substrate affinity, hydrogel architecture, and microenvironmental factors such as pH and ionic strength [172].

Altogether, these mechanistic insights are vital for designing hydrogels with tailored degradation kinetics to match the functional requirements of their intended application, be they transient biosensors, long-term drug delivery systems, or tissue-interfacing electronics.

## 6. Conclusions and Future Perspectives

Hydrogel-based thermoelectric materials and devices represent a transformative direction in the field of soft energy harvesting. Their intrinsic properties, including mechanical compliance, ionic conductivity, and biocompatibility, position them as ideal candidates for flexible, skin-integrated, and environmentally friendly power sources. This review has summarized key developments in material composition, device architecture, and performance metrics, showcasing their potential for wearable electronics and biomedical applications. Despite notable progress, significant challenges remain in scaling these technologies from laboratory prototypes to fully functional, real-world devices.

One of the primary limitations lies in the mechanical mismatch between hydrogel electrolytes and conventional electrode materials. While hydrogels can endure substantial strain without permanent deformation, most conductive electrodes lack sufficient flexibility, leading to interfacial failure under mechanical stress. This mismatch affects the overall durability and reliability of the thermoelectric system. Future research must prioritize the development of all-hydrogel thermoelectric devices in which both the electrolyte and electrode components exhibit similar mechanical properties and compatible Young’s moduli. The incorporation of nanostructured fillers, dynamic crosslinkers, and double-network architectures may further improve mechanical resilience while maintaining the softness necessary for biointegration.

From a performance standpoint, enhancing the thermoelectric power factor remains a major challenge. While hydrogel-based materials frequently demonstrate high Seebeck coefficients due to ionic transport mechanisms, their electrical conductivity often remains insufficient for meaningful power generation. Achieving concurrent improvements in both parameters requires rational design at the molecular level, including strategies such as ion–dipole interaction tuning, phase separation techniques, and the integration of mixed ionic and electronic conductors. In parallel, combining hydrogel-based thermoelectric with other energy harvesting and storage modalities, such as triboelectric, piezoelectric, or biochemical systems, offers a promising pathway to increase overall energy output and enable hybrid self-powered platforms.

Device-level considerations also play a crucial role in determining performance and practical viability. As wearable systems are subjected to continuous deformation, future designs must incorporate structures that can accommodate mechanical strain while preserving functional interfaces. Strategies such as island–bridge layouts, embedded conductive meshes, and ultra-thin films have proven effective in enhancing mechanical robustness. Simultaneously, the development of miniaturized, wireless components compatible with soft thermoelectric platforms is essential for realizing autonomous, self-powered systems. Integration with stretchable communication circuits, power management modules, and sensor arrays will support the development of multifunctional, intelligent devices.

Looking forward, several research directions should be emphasized. Material innovation is critical, particularly the development of hydrogel systems with enhanced ionic mobility, thermal stability, and environmental tolerance. The design of multifunctional hydrogels that combine thermoelectric performance with sensing, actuation, or healing capabilities will also expand their utility in diverse applications. Furthermore, progress in biocompatibility, recyclability, and eco-safe fabrication techniques will be vital for transitioning these materials toward clinical and commercial adoption.

Ultimately, hydrogel-based thermoelectric devices have the potential to reshape the landscape of wearable and implantable electronics. Their softness, adaptability, and functional tunability offer unique advantages over traditional solid-state materials. By addressing current limitations in material performance, device integration, and system-level functionality, these technologies can evolve into robust solutions for continuous health monitoring, smart textiles, and next-generation human–machine interfaces. With coordinated efforts across disciplines, hydrogel thermoelectric may soon advance from an emerging research concept to a foundational element of future bioelectronic systems.

## Figures and Tables

**Table 1:** A summary of polymer gel-based materials, their particulars for thermoelectric devices, along with their performances.

Polymer Gel Materials	Particulars	Thermopower (S) [mVK^−1^]	Electrical Conductivity (*σ*) [Sm^−1^]	Thermo Electric Power Factor (S^2^σ) [mWm^−1^ K^−2^]	Ref.
Cellulose-Benzyltrimethyl ammonium hydroxide	Controllable Seebeck coefficient	2.61	3.8	0.42	[58]
PVA-NaOH	Hydration connections and synergistic coordination to produce huge negative thermopower	−37.61	7.36 × 10^−3^	-	[59]
Poly(3,4-ethylenedioxythiophene), Ionic poly(2-acrylamido-2-methyl-1-propanesulfonic acid)	Self-healing, wearable, and transparent thermoelectric	−25.1	15.9	9.94	[60]
PVA-HCl	Achieved huge value of H+ transport	38.20	1.887	-	[61]
Polyacrylamide/poly(vinyl alcohol)/cellulose nanofiber	Outstanding performance of wearable electronics	1.69	1.68	4.79 × 10^−2^	[62]
Polyacrylamide/[Fe(CN)_6_^3−^/Fe(CN)_6_^4−^]	Stretchable thermoelectric hydrogel	4.5	9.1	2.22	[63]
PVA/[Fe(CN)_6_^3−^/Fe(CN)_6_^4−^]	Stretchable, high-strength, and quasi-solid thermocell	6.5	2.6	15.6 × 10^−2^	[64]
Tellurium-nanowire-doped poly(3,4-ethylenedioxythiophene (PEDOT): polystyrenesulfonate (PSS)/PVA	Outstanding Seebeck coefficient and high stretchability	78.7 × 10^−2^	1.5	6.81 × 10^−4^	[65]
Poly(acrylic acid)/LiCl	Outstanding capacity for self-regeneration, freezing resistance, including elevated thermoelectric characteristics	11.3	5.98	-	[66]
Gelatin/[Fe(CN)_6_^3−^/Fe(CN)_6_^4−^]/I^−^/I_3_^−^	Double sandwich structure created by combining two asymmetric gels for excellent thermoelectric performance	5.2	45.0 × 10^−2^	5.2	[67]
Polyethylene oxide/lithium bis(trifluoromethanesulfonyl)imide/ 1-Ethyl-3-methyl imidazolium chloride	Robust, self-healing ionogel with adjustable thermoelectric characteristics	13	0.3	9.7 × 10^−2^	[68]
Gelatin/polyacrylamide	Adhesion triggered by skin temperature, and detachment initiated by low temperature	10.4	8.3	0.4	[69]
Poly(acrylic acid-co-Nisopropylacrylamide) nanoparticles	Good Seebeck coefficient and extremely effective thermoelectric conversion	−9.5	2	4.8 × 10^−4^	[70]
Poly(acrylic acid/Xanthan gum/Bi_2_Se_0.3_Te_2.7_	Excellent self-healing and stretchy performance	−0.45	5	-	[71]
Polyacrylamide/polydopamine/carboxylated carbon nanotubes/polyaniline	Advanced wearable technology that can capture waste heat	18.6	17.53	6.06	[72]
PVA/Sodium alginate/polyethylene glycol/	Superb stretchy gel with a huge ionic Seebeck value	66.7	-	13.96	[73]
Poly(methyl methacrylate-co-methyl acrylate)/Mxene	Stable in the environment and mechanically adaptable	−8.8	-	2.5 × 10^−2^	[74]
Poly(vinylidene fluoride-cohexafluoropropylene)	Crucial to harness the Earth’s vast supply of low-grade thermal energy	26.1	-	≈0.46	[75]
Poly (vinylidene fluoride-co-hexafluoropropylene)/1-ethyl-3-methylimidazolium dicyanamide	Outstanding mechanical quality	22.9	-	87.026 × 10^−2^	[76]
Metal organic framework/ poly(3,4-ethylenedioxythiophene)s with poly(styrene sulfonate)	Sustainable in terms of the environment	16.2	0.03	7.6	[77]

## Data Availability

No new data were created or analyzed in this study. Data sharing is not applicable to this article.
